# Machine learning prediction models for the popularization and dissemination of medical science popularization videos

**DOI:** 10.3389/fpubh.2026.1882986

**Published:** 2026-09-18

**Authors:** Nuo Cheng, Xiu-Ling Wang, Xiao-Xue Zeng, Hui-Jun Li, Chenxuan Xu, Yan-Ning Ma, Da-Xin Gong, Yonghui Yuan, Shuang Zang, Guang-Wei Zhang

**Affiliations:** 1 Department of Gynecology, the First Hospital of China Medical University, Shenyang, China; 2 Department of Cardiology, the First Hospital of China Medical University, Shenyang, China; 3 Department of General Practice, the First Hospital of China Medical University, Shenyang, China; 4 Smart Hospital Management Department, the First Hospital of China Medical University, Shenyang, China; 5 Enduring Medicine Smart Innovation Research Institute, Shenyang, China; 6 Goizueta School of Business, Emory University, Atlanta, GA, United States; 7 The Internet Hospital Branch of the Chinese Research Hospital Association, Beijing, China; 8 Cancer Hospital of China Medical University, Liaoning Cancer Hospital & Institute, Clinical Research Center for Malignant Tumor of Liaoning Province, Shenyang, China; 9 Department of Community Nursing, School of Nursing, China Medical University, Shenyang, China

**Keywords:** health communication, machine learning, medical science popularization short videos, non-medical factors, prediction models

## Abstract

**Objective:**

To summarize the current production and release trends of medical science popularization videos, analyze the effect of non-medical factors on their spread, and develop dissemination-prediction models using machine learning (ML) algorithms.

**Methods:**

We identified a sample of medical science popularization videos on TikTok (*n* = 566), Bilibili (*n* = 50), Xiaohongshu (*n* = 54), and International TikTok (*n* = 46) platforms. Thirty six non-medical features were annotated as predictor variables, with “Thumb-Up,” “Comment,” “Share” and “Collection” as outcomes. Fifteen algorithms models were constructed for each outcome using the TikTok dataset and validated on the remaining three datasets, with model performance evaluated by area under the curve (AUC) and Brier score.

**Results:**

In the quantitative analysis of the 4 outcomes, we identified significant disparities among different videos, “Thumb-Up” with a range from 0 to 2.72 million, “Collection” with 1 to 1.36 million, “Share” with 1 to 898 thousand, and “Comment” with 0 and 200 thousand. Subsequently, four best-performing models were ultimately confirmed through internal and external validation, all of which were RF models, including “Thumb-Up” (AUC = 0.8802), “Collection” (AUC = 0.7685), “Share” (AUC = 0.7872), “Comment” (AUC = 0.8077). Weight analysis identified the video duration, video description length, and shooting in department office emerged as the most three crucial parameters across all four models.

**Conclusion:**

This study demonstrates the significant impact of non-medical factors on the dissemination of medical science popularization videos, and shows that prediction models base on these factors can effectively forecast video spread and popularity. These findings may contribute to enhancing These findings, thereby advancing health education and strengthen public health literacy.

## 1 Background

In the digital age, medical science popularization videos have become a powerful tool for widely disseminating medical knowledge and information. These videos provide the public with crucial insights into health, disease, and medical technology, thereby promoting public health literacy and behavior (1, 2). TikTok, as one of the most well-known short video platforms all over the world, is known for its short and engaging video format. TikTok boasts over 750 million daily active users in China (3). Compared to traditional information dissemination methods, short videos in TikTok have many advantages, including stronger visibility, attractiveness, interactivity and faster transmission speed, making it an emerging popular video center for medical science (4). The dissemination of these videos helps to narrow the gap between medical professionals and the public, providing short, accessible insights into complex medical topics (5).

In the dissemination process of medical science popularization videos, it is not only necessary to ensure the reliability and authenticity of the content (6, 7), but also some non-medical factors can significantly affect the dissemination and attractiveness, such as video production quality, animation, visual aids, video length, title length, and so on (8, 9). Compared to the professionalism of medical information, these non-medical factors often play an undeniable role (10). Therefore, by analyzing these non-medical factors, it is possible to predict and promote the dissemination intensity and effectiveness of medical science popularization videos. Currently, there are few studies specifically targeting these non-medical factors.

In recent times, machine learning (ML) models, including but not limited to Decision Tree (DT), Random Forest (RF), have received widespread attention due to their outstanding predictive abilities. In contrast to traditional multiple logistic regression (LR) models, ML models operate on novel logical paradigms, which can more effectively utilize data, and show enhanced predictive performance on diverse critical fields such as predicting High-risk factors, key feature variables and so on. ML has emerged as a cutting-edge technology for predictive modeling and decision-making (11, 12), providing a promising strategy for constructing influence prediction models for science popularization videos before their release. To this end, this study was designed to search medical science popularization videos on the TikTok platform, extract non-medical professional feature parameters, conduct a statistical analysis of their distribution characteristics, and subsequently develop and validate ML models for four influence outcome metrics (Thumb-Up, Comment, Share and Collection).

## 2 Methods

### 2.1 Study subjects

We used “medical science popularization” as the keyword to search on the four platforms, including TikTok^1^ from January 1, 2023 to April 1, 2026, as well as Bilibili app, Xiaohongshu app, International TikTok from June 1, 2023 to April 1, 2026. Use Chinese keyword for Chinese platforms and English ones for international TikTok. A two-stage sampling strategy was adopted, with daily searches during the initial 3 months, followed by weekly searches supplemented by event-based searches around major health-awareness days and public health campaigns.

Inclusion criteria: (1) The video published by tertiary hospitals or above; (2) The videos published by doctors with officially verified accounts; (3) The theme is related to medical science popularization, health education, prevention and treatment, and nursing. Exclusion criteria: The content is repetitive, non-original, commercial, incorrect and involves advertising. Independent review was conducted by two physicians, and any disagreements were resolved by consulting a third reviewer. Finally, 716 videos were included, in which 566 from TikTok for trend analysis and development of prediction model, 150 from Bilibili (*n* = 50), Xiaohongshu (*n* = 54) and International TikTok (*n* = 46) for validation. The video screening process is shown in Figure 1.

**Figure 1:**
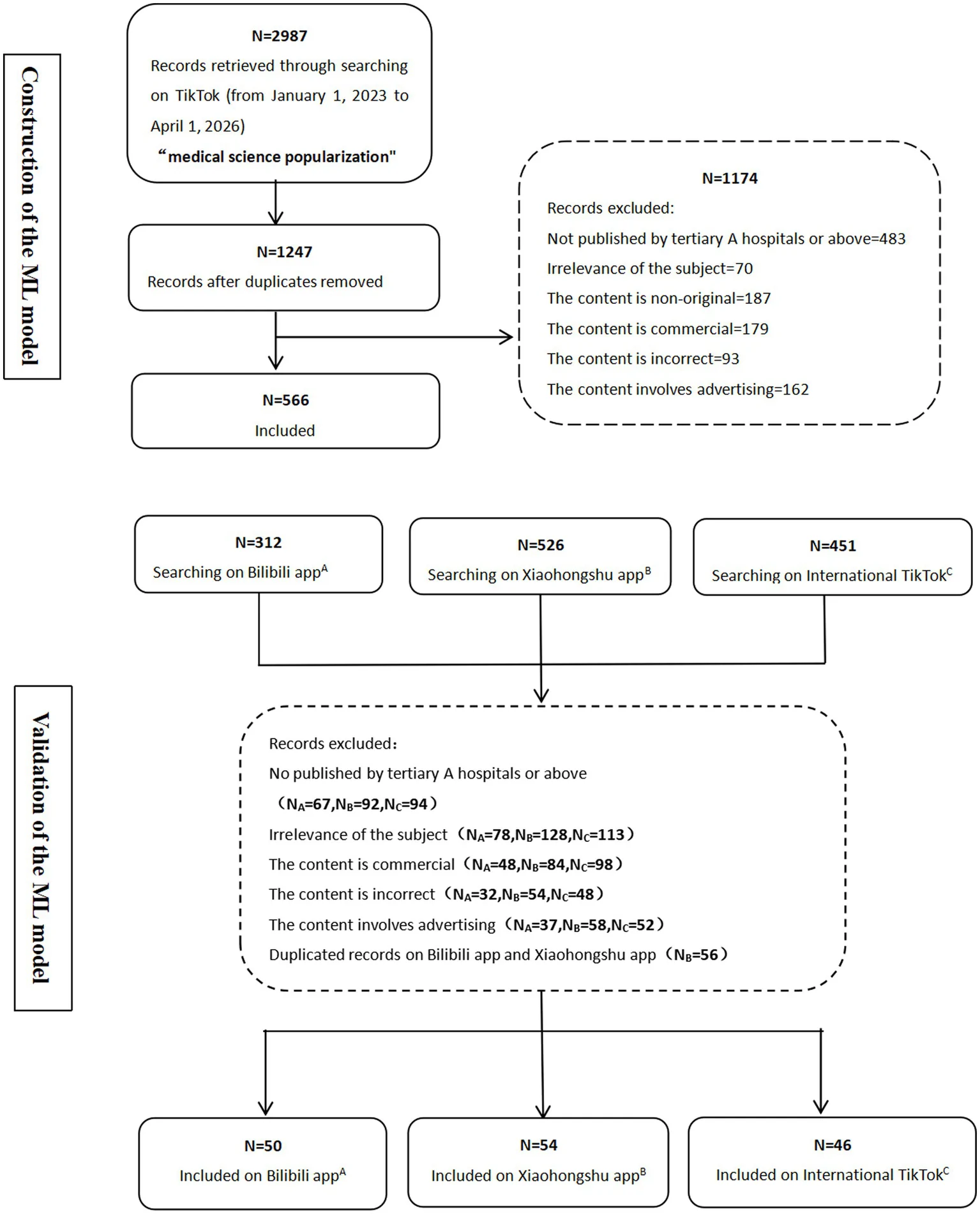
The flowchart illustrating the research process for analyzing non-medical factors of medical science popularization short videos.

### 2.2 Data collection

According previous reports (13–16), 36 factors prior to video release were identified as predictor variables, which are categorized into three aspects: host characteristics, shooting methods, production and publication (Supplementary Table 1). The factors are creator-controlled and platform-independent. We excluded factors that are mostly determined after publication and are highly platform-dependent, such as channel subscriber count, account verification status, in order to reduce the impact of such heterogeneity among different platforms. Based on prior dissemination studies and the observed pattern that engagement metrics changed most substantially within the first 3 months after publication (17, 18), we collected the three-month post-publication counts of “Thumb-Up,” “Comment,” “Share,” and “Collection” for each video as indicators of video dissemination effectiveness. Two authors (CN and WXL) who had received unified training extracted variable information from the videos according to the criteria for variable characteristics (Supplementary Table 2). After calculation of agreement and discussion with supervisor ZGW regarding inconsistencies. Inter-rater reliability was calculated by Cohen’s Kappa coefficient. The kappa values for all variables were ≥ 0.627, and all *p*-values were <0.0001 (Supplementary Table 2).

### 2.3 ML prediction models construction

The construction of ML predictive models was conducted by using the Dr. Wise multimodal research platform,^2^ which is underpinned by Python 3.6 and integrates models from the scikit-learn library (version 0.21.2). Image-related processing was performed using scikit-image (version 0.16.2) (19). The platform facilitates ML modeling through visualized configuration, encompassing variable setup, partitioning of training and testing datasets, feature engineering (feature cleaning and selection), and model hyperparameter tuning. Automated hyperparameter tuning was performed via a combination of 5-fold cross-validation and grid search, with ROC-AUC as the primary scoring metric (Supplementary Table 3). Once the parameters are configured, the platform invokes Python for model implementation and chart output. Based on the distinct distribution characteristics of the data (Supplementary Table 1), the median of each outcome was calculated and utilized as the cutoff value of binary classification, to construct four types of ML prediction models (Thumb-Up, Collection, Share and Comment). During external validation, each platform independently used its own median to divide the data into high and low groups. Fifteen classification algorithms were employed to select predictive models, including Decision Tree (DT), K Nearest Neighbors (KNN), Linear Support Vector Classifier (LSVC), Random Forest (RF), Linear Discriminant Analysis (LDA), Support Vector Machine (SVM), Gradient Boosting, Bernoulli Naive Bayes (NB), Stochastic Gradient Descent (SGD), AdaBoost, XG Boost (XGB), Gaussian Naive Bayes (NB), Logistic Regression (LR), Factorization Machine (FM) and Multilayer Perceptron (MLP).

After randomly splitting the overall dataset into training and validation sets at a ratio of 7:3 and repeating this process six times, two feature dimensionality reduction strategies were applied for each algorithmic model. The first was feature correlation analysis, for any pair with a correlation coefficient exceeding 0.9, we retained the feature that exhibited a higher absolute correlation with the target variable and excluded the other. Second, we performed L1-regularized (Lasso) logistic regression on the features surviving the correlation step. A 10-fold cross-validation approach was applied within the training set to determine the optimal penalty parameter λ, With increasing λ, the MSE first decreases and then rises, while feature coefficients are progressively shrunk to zero, allowing us to eliminate unimportant features and derive the final feature subset (Supplementary Figure 1). To avoid overfitting, all filtering thresholds (|*r*| > 0.9 and the L1 penalty) were determined exclusively from the training set, and the entire selection pipeline was nested within the cross-validation loop. Calibration was appraised using the Brier score, a Brier score of 0.25 or less signifies favorable model calibration. For model interpretability and feature importance assessment, we used SHAP (SHapley Additive exPlanations) as a post-hoc explainability method. Global feature importance was quantified by the mean absolute SHAP value, while individual SHAP values were used to visualize the direction and magnitude of feature contributions. SHAP was applied after model training and was not involved in feature selection, which was performed separately using LASSO. Thereafter, the obtained optimal models will be published on the Dr. Wise multimodal research platform at https://keyan.deepwise.com/ (Authorization login required; individuals with prediction and validation needs are encouraged to contact the corresponding author). The platform supports external validation through queuing and allows predicting the psychological state of individual subjects.

### 2.4 Statistical methods

The statistical analyses were conducted with SPSS (version 26.0, SPSS, Chicago, IL, USA) and the Dr. Wise multimodal research platform.^3^ Normally distributed data were presented as mean with standard deviation. Non-normally distributed data were expressed as medians (interquartile range [IQR]). Categorical data is represented by the number of examples and rate (%). Logistic analysis was used to screen the associated factors. A significance level of *p* < 0.05 (two-tailed) was considered to indicate statistically significant differences.

## 3 Results

### 3.1 General information description

Among 566 videos from TikTok, the minimum and maximum numbers of “Thumb-Up” are 0 and 2.72 million respectively, with a median of 31,000. The minimum and maximum numbers of “Collection” are 1 and 1.36 million respectively, with a median of 5,504. The minimum and maximum numbers of “Share” are 1 and 898 thousand respectively, with a median of 9,050. The minimum and maximum numbers of “Comment” are 0 and 200 thousand respectively, with a median of 1,058. A total of 34 variables were retained in the final models, whereas subtitles and lighting tone were excluded. In terms of host characteristics, the anchors of videos are mainly male, with chief physicians and more specialties in internal medicine. They mostly wear doctor’s gowns, with no make-up, who are expressive and have more body language. In terms of shooting techniques, videos are often shot from the front of a fixed vertical screen at close range in ambient light, with one person in the majority, often in the departmental office. In terms of video production and publication, most videos do not have background music, animation effects, beauty effects, and stickers, but have prompts and name bars. The publication time is mostly from 17:00 to 19:00 (Supplementary Table 1). There is a positive correlation among the four outcome indicators, and the difference is statistically significant (Table 1).

**Table 1:** The correlation among the four outcome indicators (*N* = 566).

Characteristics		Thumb-Up	Collection	Share	Comment
Thumb-Up	Pearson correlation (*r*)	1			
	Sig.(two-tailed)				
Collection	Pearson correlation (*r*)	0.375^**^	1		
	Sig.(two-tailed)	*p* < 0.001			
share	Pearson correlation (*r*)	0.329^**^	0.518^**^	1	
	Sig.(two-tailed)	*p* < 0.001	*p* < 0.001		
comment	Pearson correlation (*r*)	0.744^**^	0.533^**^	0.449^**^	1
	Sig.(two-tailed)	*p* < 0.001	*p* < 0.001	*p* < 0.001	

**p < 0.01.

### 3.2 Comparison of various prediction models

Based on the results of AUC comparison, we identified 8 candidate models in the preliminary stage, comprising 1 for Thumb-Up (Figure 2A), 2 for Collection (Figure 2B), 2 for Share (Figure 2C), 5 for Comment (Figure 2D). Subsequently, models whose calibration curves or decision curves failed to meet the predefined criteria were excluded (Supplementary Figure 2). The remaining five confirmed best-performing models, predicting the four outcomes, exhibited satisfactory discriminative capabilities, as supported by their respective confusion matrices and ROC curves: “Thumb-Up” RF Model (AUC = 0.8802, Figures 3A,B), “Collection” RF Model (AUC = 0.7685, Figures 4A,B), “Share” RF Model (AUC = 0.7872, Figures 5A,B), “Comment” RF Model (AUC = 0.8077, Figures 6A,B), “Comment” XGboost Model (AUC = 0.7970, Supplementary Figures 2A,B). Moreover, their Precision-Recall (P-R) curves provide additional validation to the observed good discriminative ability (the subfigures C of Figures 3–6, respectively). In the subsequent external validation phase, the “Comment” XGboost Model was excluded because it yielded Brier Scores >0.25 on all three datasets (Figure 7E). Finally, four RF models were retained.

**Figure 2:**
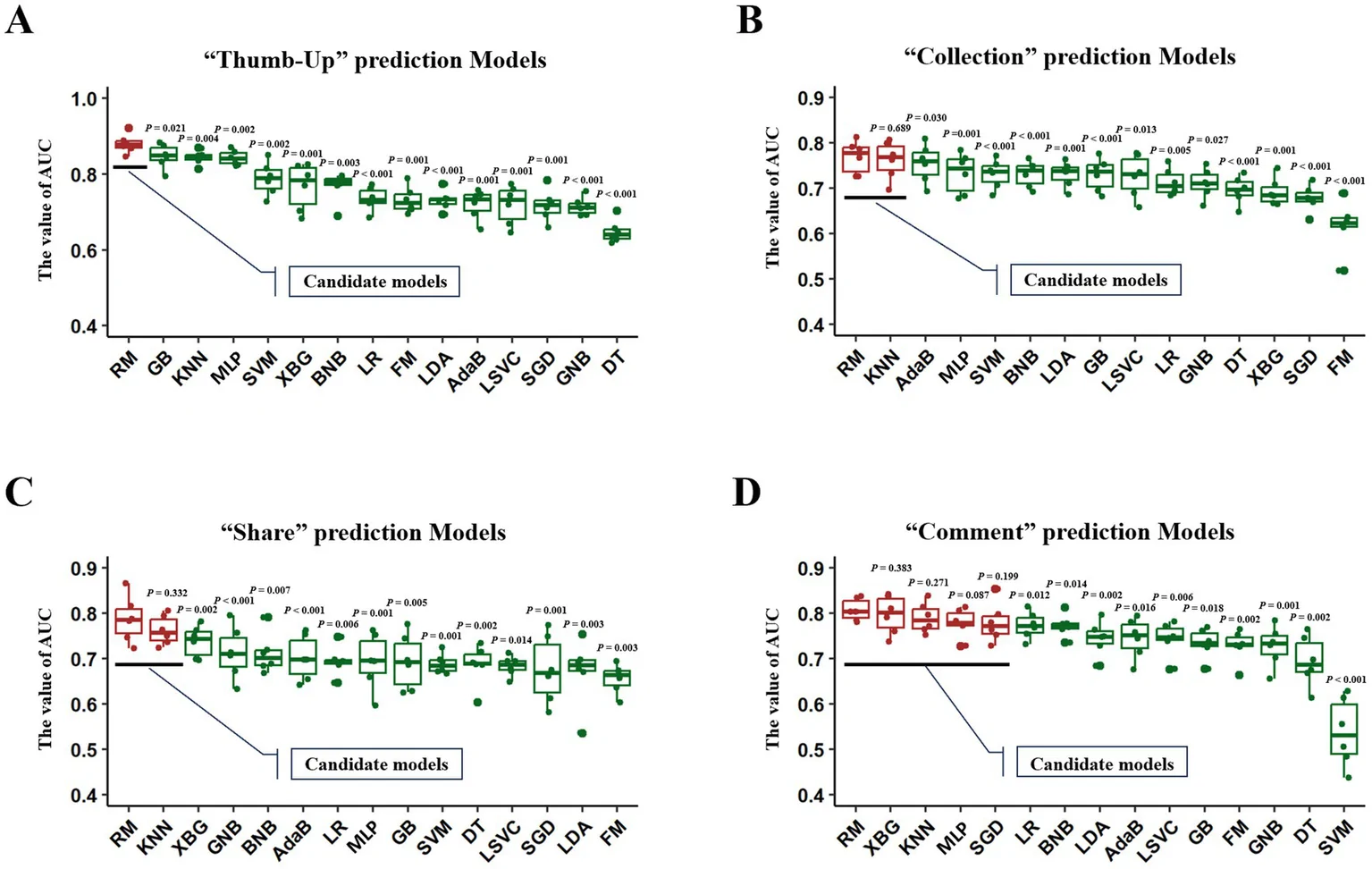
The box plot comparing the AUC values of various models was used to evaluate and select the candidate models based on their performance. AUC, area under the curve.

**Figure 3:**
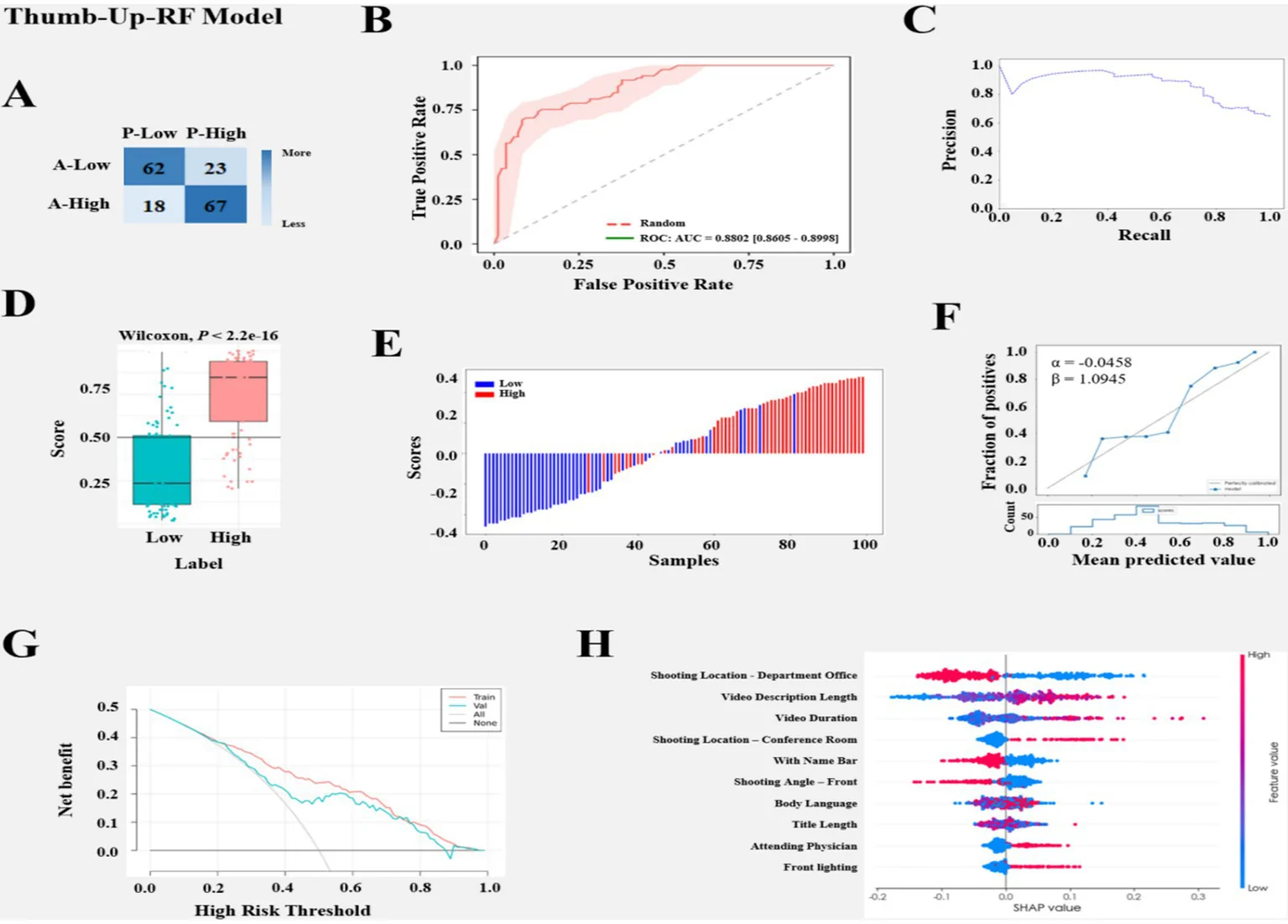
The performance of Thumb-Up RF Model. **(A)** The confusion matrix of actual and predicted quantities in low and high groups; **(B)** ROC curve in validation set; **(C)** P-R curve in validation set; **(D)** Rad scores box plot in validation set; **(E)** Probability distribution map; **(F)** Calibration plot of the predicted curve in validation set; **(G)** The decision curves in validation set; **(H)** SHAP beeswarm plot.

**Figure 4:**
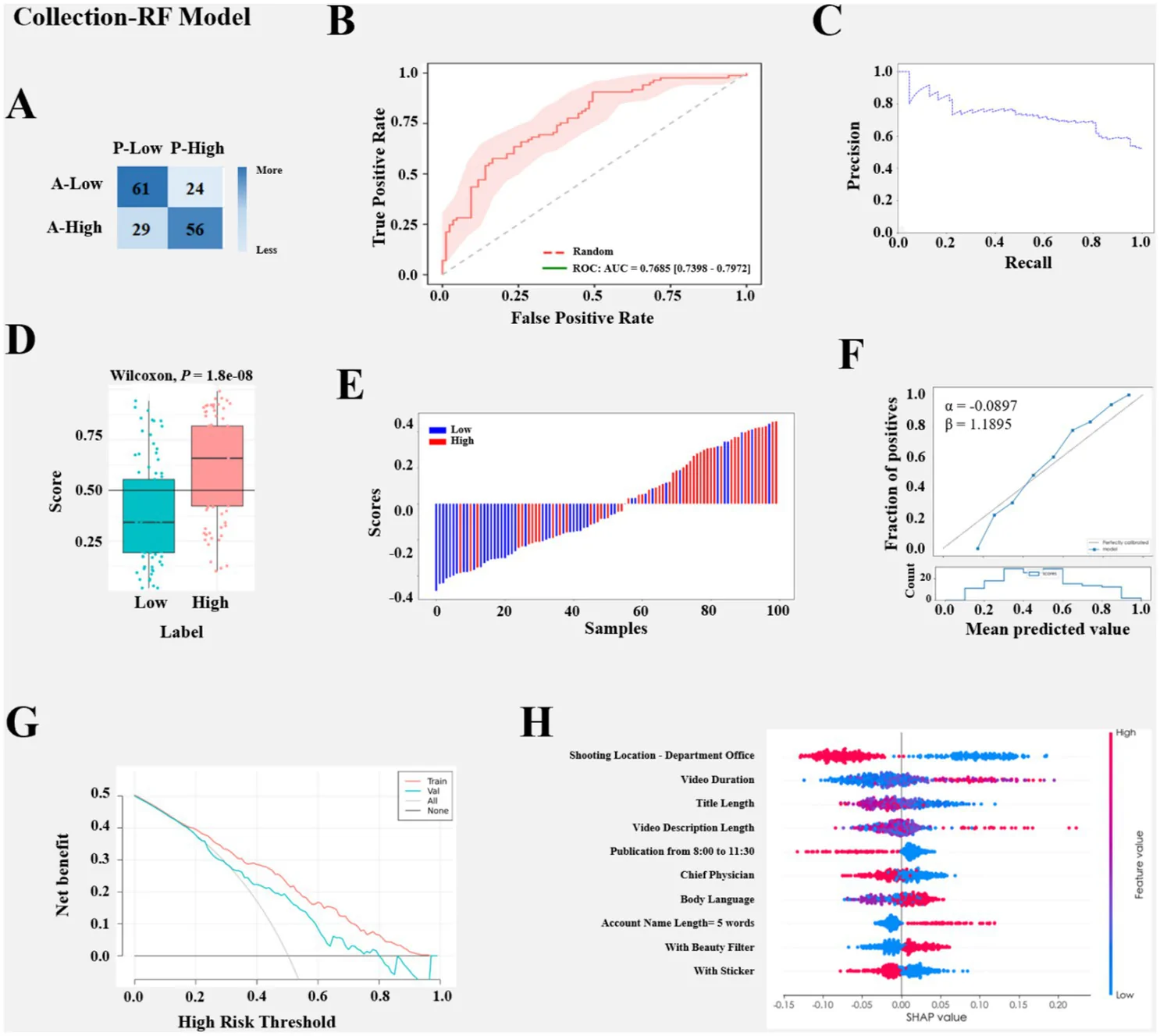
The performance of Collection-RF Model. **(A)** The confusion matrix of actual and predicted quantities in low and high groups; **(B)** ROC curve in validation set; **(C)** P-R curve in validation set; **(D)** RAD scores box plot in validation set; **(E)** Probability distribution map; **(F)** Calibration plot of the predicted curve in validation set; **(G)** The decision curves in validation set; **(H)** SHAP beeswarm plot.

**Figure 5:**
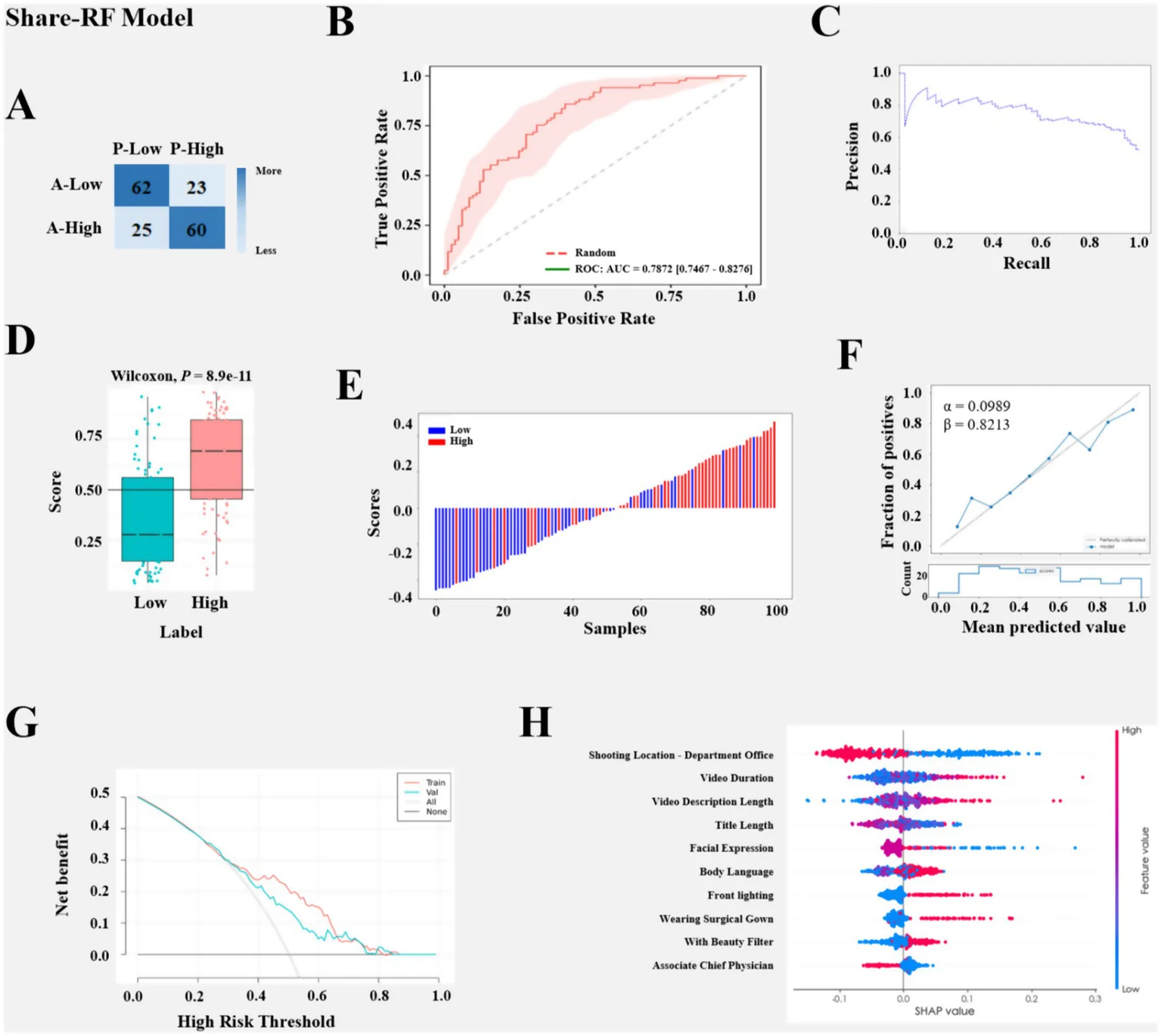
The performance of Share-RF model. **(A)** The confusion matrix of actual and predicted quantities in low and high groups; **(B)** ROC curve in validation set; **(C)** P-R curve in validation set; **(D)** Rad scores box plot in validation set; **(E)** Probability distribution map; **(F)** Calibration plot of the predicted curve in validation set; **(G)** The decision curves in validation set; **(H)** SHAP beeswarm plot.

**Figure 6:**
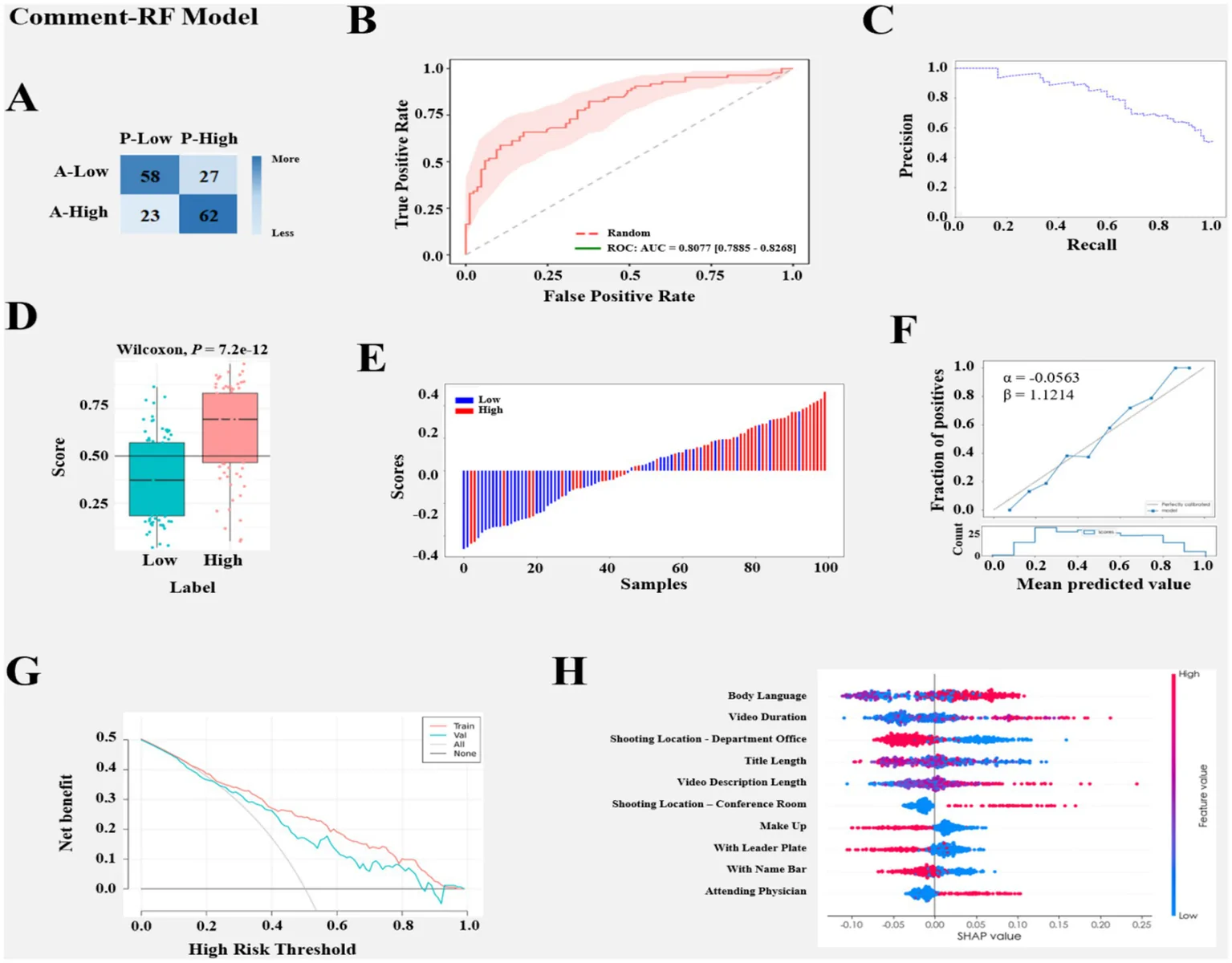
The performance of Comment-RF Model. **(A)** The confusion matrix of actual and predicted quantities in low and high groups; **(B)** ROC curve in validation set; **(C)** P-R curve in validation set; **(D)** Rad scores box plot in validation set; **(E)** Probability distribution map; **(F)** Calibration plot of the predicted curve in validation set; **(G)** The decision curves in validation set; **(H)** SHAP beeswarm plot.

**Figure 7:**
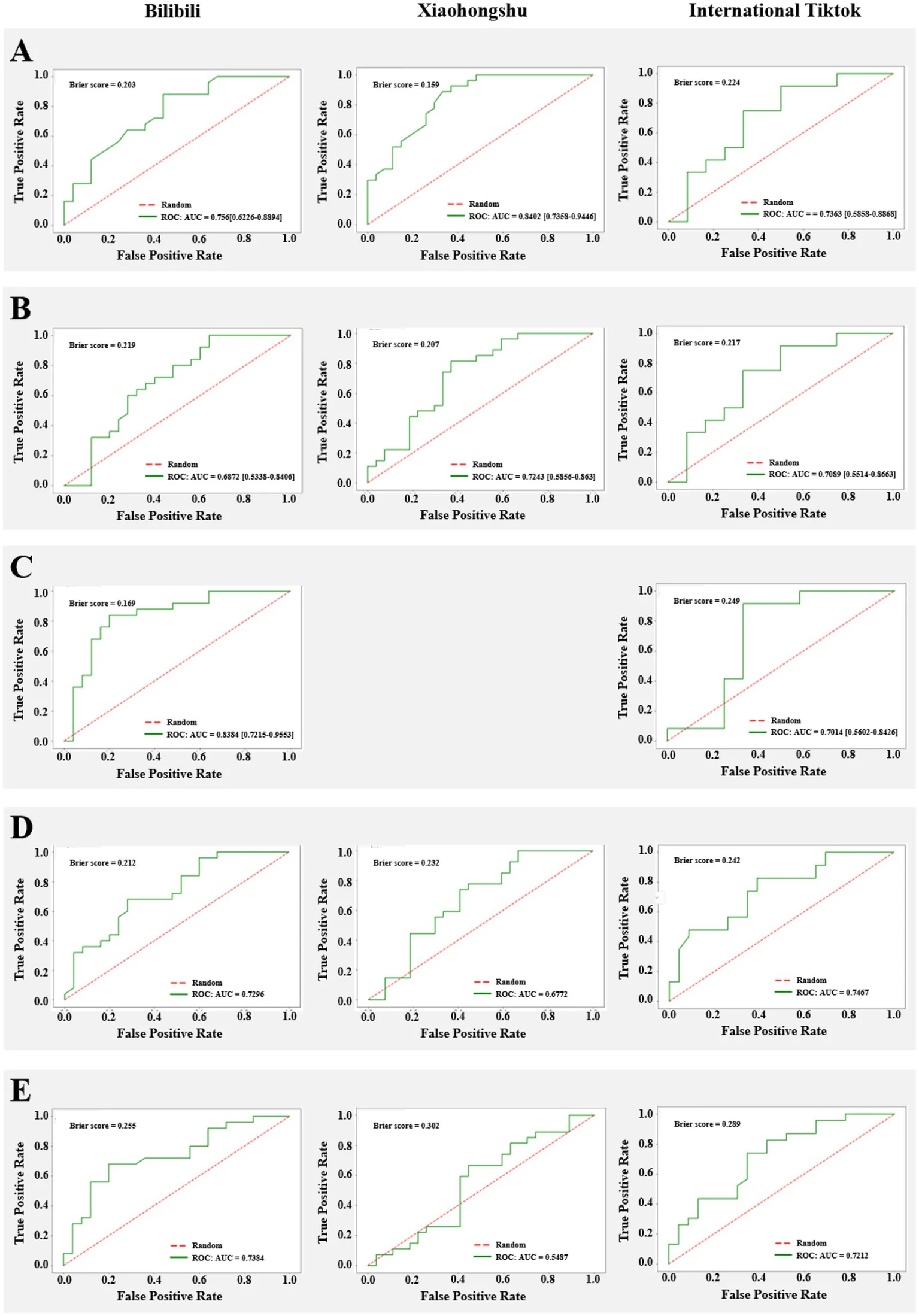
External validation performance of five models. **(A–E)** Represent, respectively, external validation ROC curves of “Thumb-Up” RF Model, “Collection-RF” Model, “Share” RF Model, “Comment” RF Model, and “Comment” RF Model, with the order of Bilibili, Xiaohongshu, and International TikTok from left to right in each line.

As depicted in Table 2, the detailed accuracy, sensitivity, specificity, precision, and F1 scores for final four models highlight their good performance in terms of a more comprehensive presentation. In terms of calibration, the good overall predictive accuracy of the four models was corroborated by their respective Brier Scores (0.140, 0.197, 0.190, 0.182, all of which are below 0.25). This affirmation was further supported by visual predictive score charts and the calibration curves (the subfigures E&F of Figures 3–6, respectively). Additionally, the decision curves have also delineated a substantial net benefit across all ML prediction models (the subfigures G of Figures 3–6, respectively). These indicate that these models have significant value in the prediction and decision-making process, and their predictive outcomes can positively influence decision-making.

**Table 2:** Analysis of predictive performance for five prediction models.

Prediction models	Performance (95% CI)								
	AUC	Accuracy	Precision	Sensitivity	F1 Score	Specificity	PPV	NPV	Brier score
Thumb-up RF model	0.8802 [0.8605–0.8998]	0.759 [0.690–0.820]	0.744 [0.680–0.810]	0.788 [0.700–0.880]	0.766 [0.700–0.830]	0.729 [0.630–0.820]	0.744 [0.650–0.830]	0.775 [0.680–0.870]	0.14
Collection RF model	0.7685 [0.7398–0.7972]	0.688 [0.620–0.760]	0.700 [0.630–0.770]	0.659 [0.560–0.760]	0.679 [0.610–0.750]	0.718 [0.620–0.810]	0.700 [0.600–0.800]	0.678 [0.580–0.770]	0.197
Share RF model	0.7872 [0.7467–0.8276]	0.718 [0.650–0.790]	0.723 [0.660–0.790]	0.706 [0.610–0.800]	0.714 [0.650–0.780]	0.729 [0.630–0.820]	0.723 [0.630–0.820]	0.713 [0.620–0.810]	0.19
Comment RF model	0.8077 [0.7885–0.8268]	0.706 [0.640–0.770]	0.697 [0.630–0.770]	0.729 [0.630–0.820]	0.713 [0.640–0.780]	0.682 [0.580–0.780]	0.697 [0.600–0.790]	0.716 [0.620–0.810]	0.182
Comment XGboost model	0.797 [0.7634–0.8306]	0.694 [0.620–0.760]	0.699 [0.630–0.770]	0.682 [0.580–0.780]	0.690 [0.620–0.760]	0.706 [0.610–0.800]	0.699 [0.600–0.800]	0.690 [0.590–0.790]	0.188

### 3.3 Parameter weight analysis results

As revealed by the analysis of parameter weights, the video duration, video description length, and shooting in the department office, emerged as the most five crucial parameters across all five models. Title length had an effect on the other three outcomes, excluding Thumb-Up. Other factors varied in importance across different models (the subfigures H of Figures 3–6, respectively). The univariate logistic regression analysis shows that the video duration, the length of videos description, male, resident, attending physician, surgical gown, distant shot can promote all outcomes, while more, serious and moderate facial expression, make-up, department office, with Leader Plate, account name length are hindrances (Supplementary Table 4). Combining the results of descriptive statistics, ML weight analysis, and logistic regression analysis, we confirmed trends in factors that promoting or impeding Thumb-Up, Collection, Share, and Comment shown in Table 3.

**Table 3:** The promoting or hindering effects of top 10 variables in RF models.

Characteristics	Thumb-Up	Collection	Share	Comment
Production and publication				
Video description length	↑*	↑*	↑*	↑*
Video duration	↑*	↑*	↑*	↑*
Title length	↓	↓*	↓*	↓*
With beauty filter		↑	↑	
With sticker		↓		
With name bar	↓*			↓
Publication from 8:00 to 11:30		↓*		
With leader plate				↓
Account name length = 5 words		↑		
Host characteristics				
Body language	↑	↑	↑	↑*
Chief physician		↓		
Associate chief physician			↓	
Attending physician	↑			↑
Wearing surgical gown			↑	
Facial expression			↓*	
Make up				↓
Shooting techniques				
Shooting Location - Department Office	↓*	↓*	↓*	↓*
Shooting Location - Conference Room	↑*			↑
Shooting Angle -Front	↓			
Shooting Light-Front light	↑		↑	

①↑ Promoting variables ↓ hindering variables; ②* Top 5 variables.

## 4 Discussion

In this study, we summarized the production and release trends of medical science popularization short videos, including host characteristics and commonly used shooting and production techniques, to enhance understanding of the dissemination of medical scientific information on this emerging communication platform. Based on these non-medical factors, for the first time, we developed and validated four ML models for predicting the influence of these videos, achieving precise predictions prior to their release. More importantly, these models identified the significance of different influencing factors, providing guidance for the entire video production process. Therefore, this study will help improve public awareness of medical knowledge by elucidating how these factors interact and collectively affect the popularity and dissemination speed of medical content. By enhancing the popularity and dissemination likelihood of these videos, our study is expected to increase the impact of medical science popularization efforts, thereby contributing to improved public health literacy.

It is necessary and meaningful to study the non-medical factors of medical education video through the vital video platforms, such as TikTok (20). More and more health care professionals are using them to convey important health information to the public, and more research focuses on the analysis of medical care professional video content and form on various platforms (13, 21–23). Du et al. (23) explored the content and quality of videos on TikTok and Bilibili platforms, and analyzed the relationship with the number of views, comments, shares, and favorites. Rudisill et al. (24) and Wang et al. (25) evaluated the reliability and educational quality of videos and found that longer video durations predicted higher scores, the former showing no independent correlation between uploaded source or content and quality score, while the latter showing that academic and clinical sources can lead to higher video quality. A study noted the influence of non-medical factors such as video style, original music by analyzing the likes, comments, and reposts of the top 100 most popular micro-videos (13). Li et al. (15) investigated how the video attributes (video format, type and content) are related to quantitative indicators of user engagement (number of views, likes, comments and shares). Although these studies suggest that non-medical factors may be key to attracting the public to watch videos, there has few studies focused exclusively on these non-medical factors. Therefore, this study controlled for consistency in professionalism and quality to observe the impact of non-medical factors on the four influence indicators. The results further directly demonstrate the critical role of non-medical factors. The significant differences in views, likes, shares, and collections between videos caused by non-medical factors suggest that these factors may have a more decisive influence than the video’s professionalism and quality. This can be primarily explained by the fact that the audience often lacks expertise in medical knowledge, making them likely to overlook medical professionalism and instead rely on non-medical factors to judge whether a popular science video is worth watching and sharing.

From a methodological perspective, analyzing video health communication is currently widely based on content analysis methods (13, 26). Some studies also use multivariate linear analysis based on traditional statistics to explore factors related to video quality and associated factors (27, 28). However, referring to the development of medical big data mining research, these methods have significant limitations in exploring the complex nonlinear relationships commonly present among data (29). In recent times, ML has emerged as a cutting-edge technology for predictive modeling and decision-making, substantially enhancing the precision of outcome predictions (30). Additionally, ML model can distinguish the importance of different variables, and avoid one-sided and subjective observation of a single indicator alone (31). Therefore, it has been widely applied in the development of predictive models across various research domains (32–34). In the field of video science popularization, ML is commonly used to analyze and evaluate public willingness (35) and emotional tendencies (36, 37), as well as to analyze video quality (38, 39). For example, by using ML to identify, retrieve, and qualify medical related and understandable YouTube videos, domain experts can then review and recommend these videos for dissemination and public education (40). Usman et al. (41) proposed that ML models can effectively predict customer satisfaction from healthcare datasets. Alghamdi et al. (42) used ML models for fake news detection, which demonstrate good predictive performance.

In this study, we adopted the application models of ML from the field of disease prediction to explore the development of influence prediction models for medical science popularization videos, comparing the performance of 15 commonly used ML algorithms. Finally, the RF algorithm outperformed all other models on the four outcome indicators. Although XGBoost exhibited robust performance on the “comments” outcome in internal validation, its external validation results were suboptimal. RF and XGboost algorithm are non-linear classifiers that can use non-linear functions and complex decision boundaries to classify data with high accuracy and robustness (43). RF employs an ensemble of a large number of decision trees built using Bagging and random feature selection strategies. Each tree recursively partitions the feature space to yield nonlinear decision boundaries, while the ensemble approach serves to reduce variance and enhance generalization capability. Consequently, without presupposing any specific functional form, RF autonomously captures complex interactions and nonlinear effects among predictor variables (44–46). RF method is very popular in ML, it is easy to train and performs well (47), which is the same as our results. Moreover, we found that nonlinear algorithms significantly outperform traditional linear algorithms (e.g., LR), further confirming the existence of complex data relationships between non-medical factors and outcomes.

After developing the machine learning models, we performed external validation using data from three other platforms. To mitigate the potential impact of differences across social media platforms in terms of user demographics, recommendation mechanisms, and engagement behaviors, we used platform-specific median values to define relative dissemination performance rather than absolute engagement counts (48). This approach was intended to reduce the interference of platform-specific scale differences during external validation, enabling the models to focus on identifying the true contribution of non-medical factors to dissemination effectiveness, rather than on platform-dependent popularity metrics. The median-based dichotomization method was adopted because it provides balanced class distributions in the absence of widely accepted clinical or practical thresholds, facilitating model training and enhancing the stability of classification algorithms (49, 50). This method has been widely employed in biomedical machine learning and clinical prediction research. In addition, all dissemination metrics were collected using a uniform three-month post-publication observation window, thereby minimizing the impact of different publication times on engagement accumulation.

We found that in terms of host characteristics, the chief physician and associate chief physician assume educational responsibilities, but the attending physician are more popular among people. Although the chief physician and associate chief physician have more experience and expertise, lower-level physicians may be more humorous and more familiar with social media platforms and digital communication strategies, their shooting methods or content more vivid and interesting. Studies have shown that humor can significantly enhance user immersion and entertainment, especially when combined with medical information, thereby enhancing user engagement (14, 51). Our research results show that people prefer to share videos posted by medical personnel wearing surgical gowns. Body language is also an added advantage in videos, as it can better promote viewer engagement and thereby facilitate video dissemination. Despite people’s desire for beauty (52), and the fact that with beauty filter aids in video collection and sharing, makeup does not facilitate comments from viewers.

In terms of shooting techniques, the shooting location and angle have a higher weight. The video shooting in the departmental office is not good, while in the conference room it is better. The former is relatively noisy, while the latter is relatively quiet, which may have resulted in superior video capture and presentation effects (53). The side and oblique shooting angles are also better than those of frontal shooting, which are natural and clear in these approaches, and therefore more popular.

In terms of video production and publication, the length of video description, duration, and length of title can all affect dissemination and engagement. People prefer videos with longer duration, detailed descriptions, and concise titles. A Research has shown that longer videos tend to provide more comprehensive information and are generally of higher quality (54). Additionally, concise and focused titles are associated with higher numbers of likes and comments (55). Sticker, name bar, and leader plate negatively affect the numbers of thumb-up, collection, and comments on videos. This may be attributable to the fact that medical videos adhere to the highest standards of rigor and seriousness, and should convey a sense of conviction and gravitas. However, the underlying reasons warrant further investigation. Videos published during the 8:00–13:30 decreases the number of collection, most people are working or busy during the day, users are more likely to invest more effort for the time-consuming activity of watching the video in the evening (56).

However, this study has certain limitations. First, the sample remains relatively small, and the results from this dataset should therefore be interpreted cautiously as preliminary external validation rather than definitive evidence of generalizability. Second, although we included as many variables as possible along with data from different time periods, we excluded indicators that are mostly determined after publication and are highly platform-dependent. Although this reduced heterogeneity across platforms, it might have certain effects on model stability. Furthermore, with the continued development of video science popularization and the growing public demand for health, new variables or changes may arise, necessitating ongoing research to improve the model’s generalizability. Third, we dichotomized the continuous outcome variables by median values, which may result in some loss of information. Subsequent studies could adopt regression-based machine learning approaches to directly predict continuous outcomes. Fourth, we integrated data from multiple platforms and obtained good ML models through internal and external validation. Although external validation can demonstrate the model’s transferability to heterogeneous real-world settings, it does not assess temporal stability. Future research could include longitudinal data to further evaluate the model’s performance over time.

## 5 Conclusion

ML models have shown superior performance in predicting non-medical factors that affect the dissemination and engagement of medical science popularization videos, and can better demonstrate the nonlinear relationships between factors. In addition to the reliability and authenticity of medical science popularization videos, the following points should also be noted. People prefer videos with longer duration, detailed descriptions, and concise titles. Videos locations should avoid department office; body language and beauty filters can be used appropriately, but excessive make-up is not recommended. The video content should be vivid, interesting, and easy to understand, while also maintaining the seriousness and rigor inherent to medical topics. This study aims to provide a reference for the future production and filming of medical science popularization videos, thereby facilitating the dissemination and engagement of the videos by the public, thus promoting medical health education and enhancing public health awareness and healthcare capabilities.

## Data Availability

The raw data supporting the conclusions of this article will be made available by the authors, without undue reservation.

## Footnotes

1
https://www.TikTok.com/

2
https://keyan.deepwise.com/

3
https://keyan.deepwise.com/
