# Supplementary material for: Machine learning prediction models for the popularization and dissemination of medical science popularization videos
Source: Front Public Health. 2026 Sep 18;14:1882986. doi: 10.3389/fpubh.2026.1882986 (PMC13630985; doi:10.3389/fpubh.2026.1882986)
Supplement: Supplementary file 1 [file Supplementary_file_1.docx]

**Supplementary Table 1. Characteristics of the data**

| Characteristics | | Samples(%) or Mean ± SD  （N=566） | High Thumb-Up  (% or Mean ± SD) | High Collection  (% or Mean ± SD) | High Share  (% or Mean ± SD) | High Comment  (% or Mean ± SD) |
| --- | --- | --- | --- | --- | --- | --- |
| **Host characteristics** | |  |  |  |  |  |
| Gender | Female | 216 (38.16) | 87 (30.74) | 96 (33.80) | 89 (31.34) | 87 (30.63) |
|  | Male | 350 (61.84) | 196 (69.26) | 188 (66.20) | 195 (68.66) | 197 (69.37) |
| Specialty | Emergency | 11 (1.94) | 10 (3.53) | 10 (3.52) | 10 (3.52) | 10 (3.52) |
|  | Internal medicine | 332 (58.66) | 166 (58.66) | 173 (60.92) | 172 (60.56) | 167 (58.80) |
|  | Dermatology | 62 (10.95) | 21 (7.42) | 19 (6.69) | 18 (6.34) | 23 (8.10) |
|  | Surgery | 108 (19.08) | 67 (23.67) | 65 (22.89) | 65 (22.89) | 69 (24.30) |
|  | Traditional Chinese Medicine | 53 (9.36) | 19 (6.71) | 17 (5.99) | 19 (6.69) | 15 (5.28) |
| Professional Title | Associate Chief physician | 146 (25.80) | 63 (22.26) | 71 (25.00) | 65 (22.89) | 65 (22.89) |
|  | Resident | 31 (5.48) | 27 (9.54) | 25 (8.80) | 25 (8.80) | 23 (8.10) |
|  | Chief physician | 268 (47.35) | 111 (39.22) | 114 (40.14) | 120 (42.25) | 116 (40.85) |
|  | Attending physician | 121 (21.38) | 82 (28.98) | 74 (26.06) | 74 (26.06) | 80 (28.17) |
| Dressing | Doctor’s gown | 405 (71.55) | 182 (64.31) | 186 (65.49) | 182 (64.08) | 180 (63.38) |
|  | Plain clothes | 68 (12.01) | 36 (12.72) | 36 (12.68) | 35 (12.32) | 37 (13.03) |
|  | Surgical gown | 93 (16.43) | 65 (22.97) | 62 (21.83) | 67 (23.59) | 67 (23.59) |
| Make-up | No | 421 (74.38) | 232 (81.98) | 225 (79.23) | 233 (82.04) | 235 (82.75) |
|  | Yes | 145 (25.62) | 51 (18.02) | 59 (20.77) | 51 (17.96) | 49 (17.25) |
| Expressiveness | No | 32 (5.65) | 23 (8.13) | 23 (8.10) | 23 (8.10) | 26 (9.15) |
|  | Yes | 534 (94.35) | 260 (91.87) | 261 (91.90) | 261 (91.90) | 258 (90.85) |
| Facial expression | Abundant | 54 (9.54) | 43 (15.19) | 42 (14.79) | 40 (14.08) | 43 (15.14) |
|  | More | 7 (1.24) | 1 (0.35) | 1 (0.35) | 2 (0.70) | 1 (0.35) |
|  | Moderate | 430 (75.97) | 197 (69.61) | 199 (70.07) | 195 (68.66) | 195 (68.66) |
|  | Serious | 75 (13.25) | 42 (14.84) | 42 (14.79) | 47 (16.55) | 45 (15.85) |
| Body language | No | 12 (2.12) | 2 (0.71) | 2 (0.70) | 2 (0.70) | 4 (1.41) |
|  | less | 145 (25.62) | 77 (27.21) | 84 (29.58) | 78 (27.46) | 75 (26.41) |
|  | Moderate | 126 (22.26) | 30 (10.60) | 31 (10.92) | 36 (12.68) | 32 (11.27) |
|  | More | 283 (50.00) | 174 (61.48) | 167 (58.80) | 168 (59.15) | 173 (60.92) |
| **Shooting techniques** | |  |  |  |  |  |
| Shooting angle | Side | 71 (12.54) | 37 (13.07) | 41 (14.44) | 39 (13.73) | 42 (14.79) |
|  | Oblique flank | 39 (6.89) | 29 (10.25) | 28 (9.86) | 29 (10.21) | 31 (10.92) |
|  | Front | 456 (80.57) | 217 (76.68) | 215 (75.70) | 216 (76.06) | 211 (74.30) |
| Shooting light | Ambient light | 462 (81.63) | 220 (77.74) | 222 (78.17) | 222 (78.17) | 227 (79.93) |
|  | Front light | 104 (18.37) | 63 (22.26) | 62 (21.83) | 62 (21.83) | 57 (20.07) |
| Shooting scence | Close shot | 532 (93.99) | 253 (89.40) | 256 (90.14) | 257 (90.49) | 253 (89.08) |
|  | Mid-shot | 15 (2.65) | 13 (4.59) | 11 (3.87) | 12 (4.23) | 13 (4.58) |
|  | Distant shot | 19 (3.36) | 17 (6.01) | 17 (5.99) | 15 (5.28) | 18 (6.34) |
| Shooting location | White wall | 58 (10.25) | 36 (12.72) | 37 (13.03) | 37 (13.03) | 36 (12.68) |
|  | Background coding | 4 (0.71) | 2 (0.71) | 2 (0.70) | 2 (0.70) | 2 (0.70) |
|  | Department | 16 (2.83) | 12 (4.24) | 12 (4.23) | 12 (4.23) | 12 (4.23) |
|  | Curtain | 12 (2.12) | 7 (2.47) | 9 (3.17) | 5 (1.76) | 6 (2.11) |
|  | Department office | 328 (57.95) | 115 (40.64) | 117 (41.20) | 122 (42.96) | 121 (42.61) |
|  | Conference office | 86 (15.19) | 68 (24.03) | 63 (22.18) | 60 (21.13) | 65 (22.89) |
|  | Bedroom | 32 (5.65) | 22 (7.77) | 24 (8.45) | 25 (8.80) | 24 (8.45) |
|  | Virtual background | 30 (5.30) | 21 (7.42) | 20 (7.04) | 21 (7.39) | 18 (6.34) |
| Light tone | Cold | 76 (13.43) | 43 (15.19) | 44 (15.49) | 44 (15.49) | 43 (15.14) |
|  | Warm | 490 (86.57) | 240 (84.81) | 240 (84.51) | 240 (84.51) | 241 (84.86) |
| Shooting position | Unfixed | 34 (6.01) | 22 (7.77) | 19 (6.69) | 20 (7.04) | 21 (7.39) |
|  | Fixed | 521 (92.05) | 254 (89.75) | 258 (90.85) | 257 (90.49) | 254 (89.44) |
|  | Moving | 11 (1.94) | 7 (2.47) | 7 (2.46) | 7 (2.46) | 9 (3.17) |
| Shooting methods | By others | 177 (31.27) | 91 (32.16) | 89 (31.34) | 92 (32.39) | 99 (34.86) |
|  | By selfie | 389 (68.73) | 192 (67.84) | 195 (68.66) | 192 (67.61) | 185 (65.14) |
| With teleprompter | No | 482 (85.16) | 238 (84.10) | 237 (83.45) | 238 (83.80) | 245 (86.27) |
|  | Yes | 84 (14.84) | 45 (15.90) | 47 (16.55) | 46 (16.20) | 39 (13.73) |
| Vertical screen | No | 11 (1.94) | 9 (3.18) | 10 (3.52) | 9 (3.17) | 9 (3.17) |
|  | Yes | 555 (98.06) | 274 (96.82) | 274 (96.48) | 275 (96.83) | 275 (96.83) |
| Number of people in video | One | 537 (94.88) | 268 (94.70) | 267 (94.01) | 268 (94.37) | 267 (94.01) |
|  | Two | 27 (4.77) | 13 (4.59) | 15 (5.28) | 14 (4.93) | 15 (5.28) |
|  | Three or more | 2 (0.35) | 2 (0.71) | 2 (0.70) | 2 (0.70) | 2 (0.70) |
| Hues | Cold | 74 (13.07) | 43 (15.19) | 44 (15.49) | 43 (15.14) | 42 (14.79) |
|  | Warm | 492 (86.93) | 240 (84.81) | 240 (84.51) | 241 (84.86) | 242 (85.21) |
| **production and publication** | |  |  |  |  |  |
| Title length | | 12.65 ± 5.07 | 12.83 ± 5.43 | 12.60 ± 5.71 | 12.74 ± 5.68 | 12.43 ± 5.56 |
| Video description length | | 13.05 ± 9.04 | 15.23 ± 9.81 | 14.26 ± 9.69 | 14.45 ± 9.80 | 15.10 ± 9.79 |
| Video duration（s） | | 83.09 ± 65.21 | 97.07 ± 75.40 | 96.88 ± 74.50 | 96.36 ± 74.74 | 96.14 ± 74.96 |
| Account name length | | 5.91 ± 1.93 | 5.61 ± 1.79 | 5.68 ± 1.87 | 5.70 ± 1.87 | 5.71 ± 1.89 |
| Personal pronouns in title | No | 489 (86.40) | 242 (85.51) | 245 (86.27) | 244 (85.92) | 245 (86.27) |
|  | Yes | 77 (13.60) | 41 (14.49) | 39 (13.73) | 40 (14.08) | 39 (13.73) |
| Personal pronouns in video description | No | 484 (85.51) | 230 (81.27) | 233 (82.04) | 234 (82.39) | 231 (81.34) |
|  | Yes | 82 (14.49) | 53 (18.73) | 51 (17.96) | 50 (17.61) | 53 (18.66) |
| Background music | No | 309 (54.59) | 158 (55.83) | 168 (59.15) | 161 (56.69) | 154 (54.23) |
|  | Yes | 257 (45.41) | 125 (44.17) | 116 (40.85) | 123 (43.31) | 130 (45.77) |
| Animation effects | No | 378 (66.78) | 192 (67.84) | 197 (69.37) | 188 (66.20) | 187 (65.85) |
|  | Yes | 188 (33.22) | 91 (32.16) | 87 (30.63) | 96 (33.80) | 97 (34.15) |
| With beauty filter | No | 299 (52.83) | 137 (48.41) | 135 (47.54) | 142 (50.00) | 145 (51.06) |
|  | Yes | 267 (47.17) | 146 (51.59) | 149 (52.46) | 142 (50.00) | 139 (48.94) |
| With sticker | No | 301 (53.18) | 148 (52.30) | 160 (56.34) | 156 (54.93) | 153 (53.87) |
|  | Yes | 265 (46.82) | 135 (47.70) | 124 (43.66) | 128 (45.07) | 131 (46.13) |
| With leader plate | No | 393 (69.43) | 224 (79.15) | 216 (76.06) | 214 (75.35) | 221 (77.82) |
|  | Yes | 173 (30.57) | 59 (20.85) | 68 (23.94) | 70 (24.65) | 63 (22.18) |
| With fixed version | No | 483 (85.34) | 233 (82.33) | 235 (82.75) | 235 (82.75) | 236 (83.10) |
|  | Yes | 83 (14.66) | 50 (17.67) | 49 (17.25) | 49 (17.25) | 48 (16.90) |
| With prompt | No | 153 (27.03) | 91 (32.16) | 93 (32.75) | 92 (32.39) | 98 (34.51) |
|  | Yes | 413 (72.97) | 192 (67.84) | 191 (67.25) | 192 (67.61) | 186 (65.49) |
| With name bar | No | 268 (47.35) | 164 (57.95) | 157 (55.28) | 152 (53.52) | 158 (55.63) |
|  | Yes | 298 (52.65) | 119 (42.05) | 127 (44.72) | 132 (46.48) | 126 (44.37) |
| Public time | 0:00-2:00 | 1 (0.18) | 0 (0.00) | 0 (0.00) | 0 (0.00) | 0 (0.00) |
|  | 4:00-6:00 | 4 (0.71) | 2 (0.71) | 3 (1.06) | 2 (0.70) | 2 (0.70) |
|  | 6:00-8:00 | 13 (2.30) | 4 (1.41) | 4 (1.41) | 4 (1.41) | 4 (1.41) |
|  | 8:00-11:30 | 107 (18.90) | 45 (15.90) | 40 (14.08) | 45 (15.85) | 41 (14.44) |
|  | 11:30-13:30 | 27 (4.77) | 17 (6.01) | 19 (6.69) | 17 (5.99) | 17 (5.99) |
|  | 13:30-17:00 | 92 (16.25) | 43 (15.19) | 45 (15.85) | 52 (18.31) | 46 (16.20) |
|  | 17:00-19:00 | 189 (33.39) | 104 (36.75) | 103 (36.27) | 98 (34.51) | 105 (36.97) |
|  | 19:00-23:59 | 131 (23.14) | 68 (24.03) | 70 (24.65) | 65 (22.89) | 69 (24.30) |

**Supplementary Table 2. Standard of the non-medical features**

| **Characteristics** | | **Standard of the non-medical features** | **Kappa value** |
| --- | --- | --- | --- |
| **Host characteristics** | |  |  |
| Gender | Female | When multiple people are present, gender is coded as the majority gender or as the gender of the primary speaker. | 1.000 |
|  | Male |  |  |
| Specialty | | (Emergency/Internal medicine /Dermatology/Surgery/Traditional Chinese Medicine)  According to video content | 0.795 |
| Professional Title | | (Associate Chief physician/Resident/Chief physician/Attending physician)  According to personal profile | 0.864 |
| Dressing | Doctor’s gown | According to the dressing of the **primary** speaker in the video | 0.878 |
|  | Plain clothes |  |  |
|  | Surgical gown |  |  |
| Make-up | Yes/No | Facial modifications have been made, including applying lipstick, eyebrow hair, etc | 0.841 |
| Expressiveness | Yes/No | No expressiveness means looking at the camera with emotionless eyes, a straight face, no smile, or a dull expression, etc | 0.783 |
| Facial expression | Abundant | Almost every sentence is accompanied by a facial expression; | 0.737 |
|  | More | degree between abundant and moderate |  |
|  | Moderate | Use appropriate, suitable facial expressions |  |
|  | Serious | Almost no facial expression, no smile |  |
| Body language | No | No gestures, eye contact, etc. | 0.828 |
|  | less | Gestures, eye contact, etc. are almost non-existent |  |
|  | Moderate | Have the right gestures and the right eye contact |  |
|  | More | Gestures, eye contact, etc. are exaggerated and expansive |  |
| **Shooting techniques** | |  |  |
| Shooting angle | Side | Face and body are about 90 degrees from the camera; | 0.911 |
|  | Oblique flank | between front and side |  |
|  | Front | Face and body toward the camera |  |
| Shooting light | Ambient light | The light is soft and does not focus on the speaker | 0.754 |
|  | Front light | The light focuses on the face of the speaker, who is very bright, the shadow of the speaker can not be seen |  |
| Shooting scence | Close shot | Only the upper body of the speaker can be seen, the speaker occupies almost the entire picture | 0.960 |
|  | Mid-shot | We can see the whole body of the speaker, and the facial expression can be seen clearly |  |
|  | Distant shot | The speaker takes up a small proportion of the picture |  |
| Shooting location | | (White wall/Background coding/Department/Curtain/Department office/Conference office/Bedroom/Virtual background)  According to the majority of the scenes shown in the video. | 0.911 |
|  |  |  |  |
| Light tone | Cold | The video tends to be green and blue, giving a cool feeling | 0.960 |
|  | Warm | The video tends to orange, yellow, giving a warm feeling |  |
| Shooting position | Unfixed | The shot is switchable | 0.934 |
|  | Fixed | Keep the shot still |  |
|  | Moving | The shot is always moving |  |
| Shooting methods | By others | The shooting is flexible and changeable, and the picture that can not be shot by oneself | 0.914 |
|  | By selfie | The viewing angle in the shot is limited, usually from the front or slightly sideways |  |
| With teleprompter | Yes/No | 1)The speaker's gaze is continuously fixed on a virtual point approximately 3–5 cm above the center of the camera lens, or on a fixed point beside the lens, with very few glances elsewhere, and the pupils remain essentially motionless.  2)The eyes show regular horizontal scanning movements back and forth.  3)The head moves very little in either the horizontal or vertical direction, the neck appears stiff, and the head is kept at a largely constant angle toward the camera.  4)The speech flow is excessively smooth and uniform, with very few filler words, slips of the tongue, or self‑corrections.  If three or more of the above conditions are met, the speaker is judged as using a teleprompter | 0.627 |
| Vertical screen | Yes/No | Judge from the video | 1.000 |
| Number of people in video | One/Two/Three or more | Judge from the video | 1.000 |
| Hues | Cold | The video tends to be green and blue, giving a cool feeling | 0.898 |
|  | Warm | The video tends to orange, yellow, giving a warm feeling |  |
| **production and publication** | |  |  |
| Title length | | Measured by Chinese character count (excluding punctuation) | / |
| Video description length | | Measured by Chinese character count (excluding punctuation) | / |
| Video duration（s） | | According to the video content, measured in seconds | / |
| Account name length | | Measured by Chinese character count (excluding punctuation) | / |
| Personal pronouns in title | | The title of the video has personal pronouns (you, I, he, she, it, you, us, they) | 0.919 |
| Personal pronouns in video description | | There are personal pronouns in the video introduction (you, I, he, she, it, you, us, they) | 0.878 |
| Background music | | There is background music in the video | 1.000 |
| Animation effects | | 1)Non‑editing special effects, such as push, pull, fade in/out, wipe, and page turn;  2)Non‑physical geometric shapes, icons, cartoon characters, etc.;  3)Text is presented with dynamic effects such as gradients, pop‑in/pop‑out, and scrolling captions.  Any one of the above items qualifies the video as containing animated effects. | 0.740 |
| With beauty filter | | Publishers have better facial flatness, are whiter and brighter | 0.898 |
| With sticker | | 1)Non‑physical graphics (e.g., circles, cartoon characters) are present on the screen;  2)Fixed‑position logos, corner marks, channel identifiers, etc., with the exclusion of name bars  Any one of the above items qualifies the video as containing animated effects. | 0.959 |
| With leader plate | | There is a lead-in video before the main content | 0.879 |
| With fixed version | | The video has been produced with a title and a cover picture | 0.940 |
| With prompt | | The video has prompts. | 0.898 |
| With name bar | | A sticker that describe the identity and name of the speaker in the video | 0.978 |
| Public time | | (0:00-2:00/4:00-6:00/6:00-8:00/8:00-11:30/11:30-13:30/13:30-17:00/17:00-19:00/19:00-23:59)  According to the time displayed on the main page | / |

**Supplementary Table 3. The hyperparameter of models**

The search ranges were defined as follows: n_estimators (number of decision trees) from 50 to 100 in steps of 1; min_samples_leaf (minimum number of samples required at a leaf node) from 1 to 20; min_samples_split (minimum number of samples required to split an internal node) from 2 to 20; criterion with candidates 'gini' and 'entropy'; max_features with candidates 'sqrt' and 'log2'; bootstrap fixed to 'True' and 'False'; and class_weight fixed to 'balanced' and 'None to address class imbalance.

| **Model** | **Hyperparameter** | **Number of predictors** |
| --- | --- | --- |
| “Thumb-Up” RF | 'max_features': 'sqrt', 'criterion': 'gini', 'min_samples_split': 3, 'class_weight': None, 'n_estimators': 49, 'bootstrap': False, 'min_samples_leaf': 1 | 34 |
| “Collection” RF | 'max_features': 'log2', 'criterion': 'entropy', 'min_samples_split': 8, 'class_weight': None, 'n_estimators': 89, 'bootstrap': True, 'min_samples_leaf': 1 | 34 |
| “Share” RF | 'max_features': 'auto', 'criterion': 'gini', 'min_samples_split': 4, 'class_weight': 'balanced', 'n_estimators': 60, 'bootstrap': False, 'min_samples_leaf': 1 | 34 |
| “Comment” RF | 'max_features': 'sqrt', 'criterion': 'entropy', 'min_samples_split': 3, 'class_weight': None, 'n_estimators': 55, 'bootstrap': True, 'min_samples_leaf': 1 | 34 |

**Supplementary Table 4. The parameters in the univariate logistic regression model**

| Characteristics | | High Thumb-Up  (% or Mean ± SD) | | High Collection  (% or Mean ± SD) | | High Share  (% or Mean ± SD) | | High Comment  (% or Mean ± SD) | |
| --- | --- | --- | --- | --- | --- | --- | --- | --- | --- |
|  | | *P* | OR | *P* | OR | *P* | OR | *P* | OR |
| **Host characteristics** | |  |  |  |  |  |  |  |  |
| Gender | Female |  |  |  |  |  |  |  |  |
|  | Male↑ | **< 0.001^***^** | **1.887(1.338, 2.663)** | **0.032^*^** | **1.451 (1.032, 2.040)** | **<0.001^***^** | **1.795 (1.273, 2.531)** | **<0.001^***^** | **1.909 (1.353, 2.694)** |
| Specialty | Emergency |  |  |  |  |  |  |  |  |
|  | Internal medicine↓ | **0.029^*^** | **0.100 (0.013, 0.790)** | **0.035^*^** | **0.109 (0.014, 0.859)** | **0.034^*^** | **0.108 (0.014, 0.849)** | **0.030^*^** | **0.101 (0.013, 0.799)** |
|  | Dermatology↓ | **0.006^**^** | **0.051 (0.006, 0.427)** | **0.004^**^** | **0.044 (0.005, 0.370)** | **0.003^**^** | **0.041 (0.005, 0.343)** | **0.009^**^** | **0.059 (0.007, 0.491)** |
|  | Surgery | 0.090 | 0.163 (0.020, 1.323) | 0.077 | 0.151 (0.019, 1.223) | 0.077 | 0.151 (0.019, 1.223) | 0.105 | 0.177 (0.022, 1.434) |
|  | Traditional Chinese Medicine↓ | **0.008^**^** | **0.056 (0.007, 0.470)** | **0.005^**^** | **0.047 (0.006, 0.399)** | **0.008^**^** | **0.056 (0.007, 0.470)** | **0.003^**^** | **0.039 (0.005, 0.336)** |
| Professional Title | Associate Chief physician |  |  |  |  |  |  |  |  |
|  | Resident↑ | **< 0.001^***^** | **8.893 (2.960, 26.715)** | **0.002^**^** | **4.401 (1.705, 11.361)** | **<0.001^***^** | **5.192 (2.010, 13.411)** | **0.004^**^** | **3.583 (1.504, 8.536)** |
|  | Chief physician | 0.733 | 0.931 (0.619, 1.401) | 0.234 | 0.782 (0.522, 1.172) | 0.96 | 1.010 (0.674, 1.516) | 0.808 | 0.951 (0.634, 1.427) |
|  | Attending physician↑ | **< 0.001^***^** | **2.770 (1.676, 4.579)** | **0.041^*^** | **1.663 (1.020, 2.712)** | **0.007^**^** | **1.962 (1.202, 3.203)** | **<0.001^***^** | **2.432 (1.477, 4.002)** |
| Dressing | Doctor’s gown |  |  |  |  |  |  |  |  |
|  | Plain clothes | 0.222 | 1.378 (0.824, 2.307) | 0.284 | 1.325 (0.792, 2.216) | 0.318 | 1.300 (0.777, 2.173) | 0.129 | 1.492 (0.891, 2.499) |
|  | Surgical gown↑ | **< 0.001^***^** | **2.844 (1.752, 4.617)** | **<0.001^***^** | **2.355 (1.467, 3.780)** | **<0.001^***^** | **3.157 (1.928, 5.171)** | **<0.001^***^** | **3.221 (1.967, 5.276)** |
| Make-up | No |  |  |  |  |  |  |  |  |
|  | Yes↓ | **< 0.001^***^** | **0.442 (0.299, 0.654)** | **0.008^**^** | **0.598 (0.408, 0.876)** | **<0.001^***^** | **0.438 (0.296, 0.647)** | **<0.001^***^** | **0.404 (0.272, 0.599)** |
| Expressiveness | No |  |  |  |  |  |  |  |  |
|  | Yes↓ | **0.014^*^** | **0.371 (0.169, 0.817)** | **0.015^*^** | **0.374 (0.170, 0.824)** | **0.015^*^** | **0.374 (0.170, 0.824)** | **<0.001^***^** | **0.216 (0.087, 0.532)** |
| Facial expression | Abundant |  |  |  |  |  |  |  |  |
|  | More↓ | **0.005^**^** | **0.043 (0.005, 0.392)** | **0.007^**^** | **0.048 (0.005, 0.435)** | **0.028^*^** | **0.140 (0.024, 0.805)** | **0.005^**^** | **0.043 (0.005, 0.392)** |
|  | Moderate↓ | **< 0.001^***^** | **0.216 (0.109, 0.431)** | **<0.001^***^** | **0.246 (0.126, 0.481)** | **<0.001^***^** | **0.290 (0.154, 0.549)** | **<0.001^***^** | **0.212 (0.107, 0.423)** |
|  | Serious↓ | **0.006^**^** | **0.326 (0.146, 0.728)** | **0.012^*^** | **0.364 (0.166, 0.799)** | **0.174** | **0.588 (0.273, 1.266)** | **0.020^*^** | **0.384 (0.171, 0.860)** |
| Body language | No |  |  |  |  |  |  |  |  |
|  | Less↑ | **0.029^*^** | **5.662 (1.198, 26.751)** | **0.015^*^** | **6.885 (1.456, 32.557)** | **0.026*** | **5.821 (1.232, 27.505)** | 0.230 | 2.143 (0.618, 7.432) |
|  | Moderate | 0.578 | 1.563 (0.324, 7.530) | 0.541 | 1.632 (0.339, 7.853) | 0.386 | 2.000 (0.417, 9.581) | 0.552 | 0.681 (0.192, 2.414) |
|  | More↑ | **0.008^**^** | **7.982 (1.716, 37.119)** | **0.012^*^** | **7.198 (1.548, 33.462)** | **0.011^*^** | **7.304 (1.571, 33.957)** | 0.066 | 3.145 (0.925, 10.695) |
| **Shooting techniques** | |  |  |  |  |  |  |  |  |
| Shooting angle | Side |  |  |  |  |  |  |  |  |
|  | Oblique flank↑ | **0.025^*^** | **2.665 (1.132, 6.275)** | 0.147 | 1.863 (0.803, 4.321) | **0.048^*^** | **2.379 (1.010, 5.609)** | **0.034^*^** | **2.676 (1.077, 6.647)** |
|  | Front | 0.478 | 0.834 (0.506, 1.376) | 0.098 | 0.653 (0.394, 1.082) | 0.237 | 0.738 (0.447, 1.220) | **0.045*** | **0.595 (0.358, 0.988)** |
| Shooting light | Ambient light |  |  |  |  |  |  |  |  |
|  | Front light↑ | **0.018^*^** | **1.690 (1.096, 2.608)** | **0.034^*^** | **1.596 (1.036, 2.459)** | **0.034^*^** | **1.596 (1.036, 2.459)** | 0.296 | 1.256 (0.819, 1.924) |
| Shooting scence | Close shot |  |  |  |  |  |  |  |  |
|  | Mid-shot↑ | **0.010^**^** | **7.168 (1.602, 32.074)** | 0.066 | 2.965 (0.932, 9.429) | **0.026^*^** | **4.280 (1.194, 15.341)** | **0.010^**^** | **7.168 (1.602, 32.074)** |
|  | Distant shot↑ | **0.003^**^** | **9.374 (2.145, 40.971)** | **0.003^**^** | **9.164 (2.097, 40.055)** | **0.015^*^** | **4.013 (1.315, 12.249)** | **0.004^**^** | **19.850 (2.631, 149.757)** |
| Shooting location | White wall |  |  |  |  |  |  |  |  |
|  | Background coding | 0.635 | 0.611 (0.080, 4.655) | 0.585 | 0.568 (0.074, 4.329) | 0.585 | 0.568 (0.074, 4.329) | 0.635 | 0.611 (0.080, 4.655) |
|  | Department | 0.342 | 1.833 (0.525, 6.397) | 0.405 | 1.703 (0.487, 5.954) | 0.405 | 1.703 (0.487, 5.954) | 0.342 | 1.833 (0.525, 6.397) |
|  | Curtain | 0.809 | 0.856 (0.242, 3.029) | 0.46 | 1.703 (0.415, 6.989) | 0.162 | 0.405 (0.114, 1.438) | 0.44 | 0.611 (0.175, 2.132) |
|  | Department office↓ | **< 0.001^***^** | **0.330 (0.185, 0.587)** | **<0.001^***^** | **0.315 (0.176, 0.563)** | **<0.001^***^** | **0.336 (0.188, 0.601)** | **<0.001^***^** | **0.357 (0.201, 0.635)** |
|  | Conference office↑ | **0.027^*^** | **2.309 (1.099, 4.851)** | 0.228 | 1.555 (0.759, 3.186) | 0.454 | 1.310 (0.646, 2.654) | 0.084 | 1.892 (0.918, 3.899) |
|  | Bedroom | 0.527 | 1.344 (0.538, 3.362) | 0.279 | 1.703 (0.650, 4.460) | 0.164 | 2.027 (0.750, 5.480) | 0.216 | 1.833 (0.702, 4.788) |
|  | Virtual background | 0.461 | 1.426 (0.555, 3.665) | 0.789 | 1.135 (0.448, 2.874) | 0.561 | 1.324 (0.514, 3.414) | 0.85 | 0.917 (0.372, 2.261) |
| Light tone | Cold |  |  |  |  |  |  |  |  |
|  | Warm | 0.219 | 0.737 (0.453, 1.199) | 0.15 | 0.698 (0.428, 1.138) | 0.15 | 0.698 (0.428, 1.138) | 0.231 | 0.743 (0.456, 1.209) |
| Shooting position | Unfixed |  |  |  |  |  |  |  |  |
|  | Fixed | 0.076 | 0.519 (0.252, 1.070) | 0.473 | 0.774 (0.385, 1.557) | 0.286 | 0.681 (0.337, 1.378) | 0.145 | 0.589 (0.289, 1.201) |
|  | Moving | 0.949 | 0.955 (0.232, 3.932) | 0.651 | 1.382 (0.340, 5.616) | 0.777 | 1.225 (0.300, 4.995) | 0.232 | 2.786 (0.519, 14.963) |
| Shooting methods | By others |  |  |  |  |  |  |  |  |
|  | By selfie | 0.650 | 0.921 (0.645, 1.314) | 0.973 | 0.994 (0.697, 1.418) | 0.563 | 0.900 (0.631, 1.285) | 0.065 | 0.714 (0.500, 1.021) |
| With teleprompter | No |  |  |  |  |  |  |  |  |
|  | Yes | 0.478 | 1.183 (0.743, 1.882) | 0.252 | 1.313 (0.824, 2.093) | 0.363 | 1.241 (0.779, 1.976) | 0.457 | 0.838 (0.527, 1.334) |
| Vertical screen | No |  |  |  |  |  |  |  |  |
|  | Yes↓ | 0.052 | 0.217 (0.046, 1.012) | **0.027^*^** | **0.098 (0.012, 0.767)** | 0.053 | 0.218 (0.047, 1.019) | 0.053 | 0.218 (0.047, 1.019) |
| Number of people in video | One |  |  |  |  |  |  |  |  |
|  | Two | 0.858 | 0.932 (0.430, 2.020) | 0.555 | 1.264 (0.581, 2.751) | 0.844 | 1.081 (0.499, 2.343) | 0.555 | 1.264 (0.581, 2.751) |
|  | Three or more | 0.981 | 2126083.634 (0.000, Inf) | 0.981 | 2141979.752 (0.000, Inf) | 0.981 | 2126083.634 (0.000, Inf) | 0.981 | 2141979.752 (0.000, Inf) |
| Hues | Cold |  |  |  |  |  |  |  |  |
|  | Warm | 0.136 | 0.687 (0.419, 1.126) | 0.088 | 0.649 (0.395, 1.067) | 0.145 | 0.692 (0.422, 1.135) | 0.226 | 0.738 (0.451, 1.207) |
| **production and publication** | |  |  |  |  |  |  |  |  |
| Title length | | 0.388 | 1.014 (0.982, 1.048) | 0.808 | 0.996 (0.964, 1.029) | 0.674 | 1.007 (0.975, 1.040) | 0.291 | 0.983 (0.951, 1.015) |
| Video description length↑ | | **< 0.001^***^** | **1.060 (1.038, 1.082)** | **0.001^**^** | **1.031 (1.012, 1.051)** | **<0.001^***^** | **1.036 (1.017, 1.056)** | **<0.001^***^** | **1.056 (1.035, 1.078)** |
| Video duration（s）↑ | | **< 0.001^***^** | **1.008 (1.004, 1.011)** | **<0.001^***^** | **1.007 (1.004, 1.010)** | **<0.001^***^** | **1.007 (1.004, 1.010)** | **<0.001^***^** | **1.007 (1.004, 1.010)** |
| Account name length↓ | | **< 0.001^***^** | **0.846 (0.774, 0.925)** | **0.004^**^** | **0.879 (0.806, 0.960)** | **0.009^**^** | **0.890 (0.816, 0.971)** | **0.013^*^** | **0.895 (0.821, 0.977)** |
| Personal pronouns in title | No |  |  |  |  |  |  |  |  |
|  | Yes | 0.540 | 1.162 (0.718, 1.881) | 0.929 | 1.022 (0.632, 1.653) | 0.738 | 1.086 (0.671, 1.756) | 0.929 | 1.022 (0.632, 1.653) |
| Personal pronouns in video description | No |  |  |  |  |  |  |  |  |
|  | Yes↑ | **0.005^**^** | **2.018 (1.241, 3.283)** | **0.020^*^** | **1.772 (1.096, 2.866)** | **0.036^*^** | **1.669 (1.035, 2.693)** | **0.005^**^** | **2.002 (1.230, 3.256)** |
| Background music | No |  |  |  |  |  |  |  |  |
|  | Yes↓ | 0.555 | 0.905 (0.650, 1.260) | **0.029^*^** | **0.690 (0.495, 0.963)** | 0.315 | 0.844 (0.606, 1.175) | 0.86 | 1.030 (0.740, 1.434) |
| Animation effects | No |  |  |  |  |  |  |  |  |
|  | Yes | 0.592 | 0.909 (0.640, 1.290) | 0.191 | 0.791 (0.557, 1.124) | 0.766 | 1.055 (0.743, 1.496) | 0.634 | 1.089 (0.767, 1.545) |
| With beauty filter | No |  |  |  |  |  |  |  |  |
|  | Yes↑ | **0.036^*^** | **1.427 (1.024, 1.987)** | **0.012^*^** | **1.534 (1.101, 2.138)** | 0.177 | 1.256 (0.902, 1.748) | 0.397 | 1.153 (0.829, 1.605) |
| With sticker | No |  |  |  |  |  |  |  |  |
|  | Yes | 0.674 | 1.074 (0.772, 1.494) | 0.131 | 0.775 (0.557, 1.079) | 0.403 | 0.868 (0.624, 1.208) | 0.74 | 0.946 (0.680, 1.316) |
| With leader plate | No |  |  |  |  |  |  |  |  |
|  | Yes↓ | **< 0.001^***^** | **0.390 (0.269, 0.567)** | **<0.001^***^** | **0.531 (0.369, 0.764)** | **0.002^**^** | **0.568 (0.396, 0.817)** | **<0.001^***^** | **0.446 (0.308, 0.644)** |
| With fixed version | No |  |  |  |  |  |  |  |  |
|  | Yes↑ | **0.045^*^** | **1.626 (1.012, 2.613)** | 0.082 | 1.521 (0.948, 2.439) | 0.082 | 1.521 (0.948, 2.439) | 0.132 | 1.435 (0.896, 2.298) |
| With prompt | No |  |  |  |  |  |  |  |  |
|  | Yes↓ | **0.006^**^** | **0.592 (0.406, 0.862)** | **0.002^**^** | **0.555 (0.380, 0.810)** | **0.004^**^** | **0.576 (0.395, 0.840)** | **<0.001^***^** | **0.460 (0.314, 0.674)** |
| With name bar | No |  |  |  |  |  |  |  |  |
|  | Yes↓ | **< 0.001^***^** | **0.422 (0.301, 0.591)** | **<0.001^***^** | **0.525 (0.376, 0.734)** | **0.003^**^** | **0.607 (0.435, 0.846)** | **<0.001^***^** | **0.510 (0.365, 0.713)** |
| Public time | 0:00-2:00 |  |  |  |  |  |  |  |  |
|  | 4:00-6:00 | 0.987 | 2118180.003 (0.000, Inf) | 0.986 | 6354539.248 (0.000, Inf) | 0.98 | 779233.867 (0.000, Inf) | 0.987 | 2118179.976 (0.000, Inf) |
|  | 6:00-8:00 | 0.988 | 941413.334 (0.000, Inf) | 0.988 | 941413.222 (0.000, Inf) | 0.981 | 346326.163 (0.000, Inf) | 0.988 | 941413.323 (0.000, Inf) |
|  | 8:00-11:30 | 0.987 | 1537388.712 (0.000, Inf) | 0.987 | 1264584.925 (0.000, Inf) | 0.98 | 565572.968 (0.000, Inf) | 0.987 | 1315839.076 (0.000, Inf) |
|  | 11:30-13:30 | 0.986 | 3600906.004 (0.000, Inf) | 0.986 | 5030676.904 (0.000, Inf) | 0.979 | 1324697.575 (0.000, Inf) | 0.986 | 3600905.959 (0.000, Inf) |
|  | 13:30-17:00 | 0.987 | 1858811.023 (0.000, Inf) | 0.987 | 2028044.441 (0.000, Inf) | 0.979 | 1013004.028 (0.000, Inf) | 0.987 | 2118179.976 (0.000, Inf) |
|  | 17:00-19:00 | 0.987 | 2591655.533 (0.000, Inf) | 0.987 | 2536889.700 (0.000, Inf) | 0.98 | 839174.934 (0.000, Inf) | 0.987 | 2647724.970 (0.000, Inf) |
|  | 19:00-23:59 | 0.987 | 2286289.527 (0.000, Inf) | 0.987 | 2430698.073 (0.000, Inf) | 0.98 | 767427.294 (0.000, Inf) | 0.987 | 2357329.328 (0.000, Inf) |

① **P*＜0.05	***P*＜0.01	****P*＜0.001	② ↑ Promoting factors	↓ hindering factors
