# Supplementary material for: Machine learning prediction models for the popularization and dissemination of medical science popularization videos
Source: Front Public Health. 2026 Sep 18;14:1882986. doi: 10.3389/fpubh.2026.1882986 (PMC13630985; doi:10.3389/fpubh.2026.1882986)
Supplement: Supplementary file 2 [file Supplementary_file_2.docx]

**Supplementary Figure 1 Cross-validation MSE vs.** λ**, with the optimal** λ **highlighted**

**Supplementary Figure 2 Excluded prediction Models**

**Supplementary Figure 3 The performance of Comment-XGboost Model.**

**A.**The confusion matrix of actual and predicted quantities in low and high groups; **B.** ROC curve in validation set; **C.** P-R curve in validation set; **d**) Rad scores box plot in validation set; **E.**Probability distribution map; **F.** Calibration plot of the predicted curve in validation set; **G.** The decision curves in validation set. **H.** SHAP beeswarm plot.
